# Let-7i-5p Regulation of Cell Morphology and Migration Through Distinct Signaling Pathways in Normal and Pathogenic Urethral Fibroblasts

**DOI:** 10.3389/fbioe.2020.00428

**Published:** 2020-05-14

**Authors:** Kaile Zhang, Ranxin Yang, Jun Chen, Er Qi, Shukui Zhou, Ying Wang, Qiang Fu, Rong Chen, Xiaolan Fang

**Affiliations:** ^1^The Department of Urology, Affiliated Sixth People’s Hospital, Shanghai Jiao Tong University, Shanghai, China; ^2^Shanghai Eastern Institute of Urologic Reconstruction, Shanghai, China; ^3^Shanghai Xuhui District Xietu Street Community Health Service Center, Shanghai, China

**Keywords:** let-7i-5p, microRNA, cell migration, cell morphology, fibroblast

## Abstract

microRNAs regulate subcellular functions through distinct molecular mechanisms. In this study, we used normal and pathogenic fibroblasts in pelvic fracture urethral distraction defects (PFUDD) patients. PFUDD is a common disease that could severely affect patients’ life quality, yet little is known about the molecular mechanism associated with pathogenic fibrosis in PFUDD. Our data showed that let-7i-5p performs a multi-functional role in distinct signaling transduction pathways involved in cell morphology and cell migration in both normal and pathogenic fibroblasts. By analyzing the molecular mechanism associated with its functions, we found that let-7i-5p regulates through its direct target genes involved in collagen metabolism, cell proliferation and differentiation, TGF-beta signaling, DNA repair and ubiquitination, gene silencing and oxygen homeostasis. We conclude that let-7i-5p plays an essential role in regulating cell shape and tissue elasticity, cell migration, cell morphology and cytoskeleton, and could serve as a potential target for clinical treatment of urethral stricture patients.

## Introduction

Pelvic fracture urethral distraction defects (PFUDD) is a common disease that could severely affects patients’ life quality, largely due to excessive fibrosis and associated urethral stricture (Zhang et al., 2018). The current incidence of PFUDD is noted to be variable, usually between 5 and 25% of pelvic fractures, with a frequency of 0.32–5/100,000 for men and 0.46–7.25/100,000 for women (Alwaal et al., 2015; Barratt et al., 2018; Dixon et al., 2018). Pelvic fractures resulting in PFUDD has mortality rates between 5 and 33% (Barratt et al., 2018). Fibrosis is a key factor responsible for pathologic changes related to urethral stricture (in both primary or recurrent diseases; Zhao et al., 2018). Over the last few decades, microRNAs and their regulation of fibrosis have been studied in many specific organs, such as liver, heart, skin, kidney, and lung (Jiang et al., 2010, 2017; Vettori et al., 2012; Zhu et al., 2013; Rajasekaran et al., 2015; Li et al., 2016; O’Reilly, 2016; Bagnato et al., 2017). The major interests are in miR-29 and TGF-beta signaling pathway, focusing on their role of molecular regulation of fibrosis and/or associated excessive extracellular matrix deposition (Rajasekaran et al., 2015; O’Reilly, 2016). miRNAs in specific diseases, such as idiopathic pulmonary fibrosis (IPF), together with their functions in epithelial-mesenchymal transition (EMT) and trans-differentiation, have also been studied (Li et al., 2016). Thus, it would be extremely helpful to further understand molecular mechanisms and related miRNA signaling involved in PFUDD-associated fibrosis, in order to discover novel targets to prevent PFUDD by suppressing urethral stricture.

Our group recently performed molecular profiling of microRNAs in PFUDD patients and summarized a few candidate genes that may serve regulatory functions in fibrosis (Zhang et al., 2018). We found that miR-29 expression is moderate in normal and pathogenic scar tissues in PFUDD patients, and that expression of hsa-miR-29b-3p and hsa-miR-29c-3p were both slightly downregulated in scar tissues (0.64 vs. 0.69, scar vs. normal) from PFUDD patients (Zhang et al., 2018). Interestingly, let-7i-5p expression appeared to be one of the highest among all the microRNAs, and its expression showed an increase in scar tissue comparing to normal tissue (Zhang et al., 2018). Based on raw counts, the expression of global miRNA in normal tissue is 1,522 ± 488 (mean ± standard error), and in scar tissue it is 1,512 ± 483 (mean ± standard error). For let-7i-5p, the expression is 57,325 in normal tissues and 76,083 in scar tissues. Given its impressive abundance in normal and pathogenic fibroblasts, we hypothesize that let-7i-5p may serve as an important regulator in cellular events. Thus, we extended our work of microRNA analysis in PFUDD and discovered let-7i-5p as a novel regulator in multiple cellular events in normal and pathogenic urethral tissues. By up- or down-regulating let-7i-5p in normal human fibroblasts and pathogenic tissues, we evaluated the expression of possible molecular targets involved in those cellular functions (COL1A1, COL3A1, ELN, MMP1, VIM, FN1, ACTIN, TGFBR1, and TIMP1). Our data confirmed that let-7i-5p regulates those cellular events in distinct signaling pathways and the multi-functional regulations are through corresponding downstream target genes. In conclusion, let-7i-5p plays an essential role in regulating cell shape and tissue elasticity, cell migration, cell morphology and cytoskeleton, and it could serve as a potential biomarker and therapeutic target for clinical treatment in PFUDD patients.

## Materials and Methods

### Urethral Scar Samples

The study was approved by the Ethics Committee of Shanghai Sixth People’s Hospital. Consents were obtained from all of the patients to use their samples in scientific research. Scar tissues in urethra (Human scar fibroblasts, or HSF) were harvested from PFUDD patients undergoing urethroplasty (*n* = 5). All five subjects are males (gender ratio is 100% male) with ages ranging from 16 to 59. Patients’ baseline information was summarized in Table 1. The etiology of patients with urethral stricture was PFUDD. All the participating patients underwent primary surgery. The mean length of stricture is 1.5 cm and the locations were all at the membranous segment of urethra. Samples were harvested after surgery, sectioned and stored at –80°C until the process of RNA extraction.

**TABLE 1:** Patient baseline characteristics.

**Number of subject**	**Age**	**Gender**	**Health status**
1	59	Male	PFUDD
2	50	Male	PFUDD
3	16	Male	PFUDD
4	43	Male	PFUDD
5	44	Male	PFUDD

### Human Cell Line

Normal human foreskin fibroblasts (HFF, Catalog# SCSP-106) were provided by Stem Cell Bank, Chinese Academy of Sciences.

### Cell Transfection

HSF and HFF cells were growing in 10 cm dishes in Dulbecco’s MEM (DMEM, Gibco, Cat.# 12100-046, Carlsbad, CA, United States) supplemented with Fetal Bovine Serum (FBS) (Gibco, Cat.#10099-141, Carlsbad, CA, United States) to 10% by volume and Penicillin-Streptomycin (100 μ/mL) (Gibco, Cat.# 15140122, Carlsbad, CA, United States). 80–90% confluency cells were then detached using Trypsin (Gibco, Cat.# 25200056) and plated at 1 × 10^5^ cells/mL in 6-well dishes (2 mL/well), and incubate at 37°C overnight. The cells were transfected with lentiviral constructs (empty control construct, customized lenti-KD miRNA and lenti-OE miRNA from GENECHEM, Shanghai, China) to overexpress or knock down of let-7i-5p according to the manufacturer’s protocol. Transfection mixture was replaced by 2 mL DMEM (10% FBS) medium after 12 h. Transfected cells were grown for 72 h before imaging.

### Imaging

To confirm stable expression of each transfected construct, GFP expression of transfected cells was observed and evaluated by an Olympus IX70 microscope under fluorescent channel. For regular bright field imaging (for Transwell assay), samples were imaged with the Olympus IX70 microscope under bright light channel.

### Cell Migration Assay

Inserts with 8 μM pore size (Corning-Costar, Lowell MA) were used with matching 24-well transwell chambers. Cells were suspended in serum-free DMEM medium and adjusted to 2 × 10^5^ cells/mL. 100–150 μL cell suspension were placed in the upper chambers. The lower chambers were filled with DMEM medium with 10% FBS (600–800 μL/well). Cells were incubated at 37C for 24 h, the inserts were removed and inner side was wiped with cotton swaps. The inserts were then fixed in methanol for 30 min at room temperature, and stained with crystal violet solution (Cat.#A100528-0025, Sangon Biotech Shanghai, China) for 15–30 min and were peeled off after washing and mounted on the slides. The migrated cells were imaged with an OLYMPUS IX70 microscope using bright light channel. Six cell [HFF (NC (negative control)/SI ((for siRNA-led knockdown)/OE (overexpression), or FNC/FSI/FOE and HSF (NC (negative control)/SI ((for siRNA-led knockdown)/OE (overexpression), or SNC/SSI/SOE] were analyzed and triplicate experiments were done for all the cell types.

### Reverse Transcription (RT)-qPCR for miRNAs and Targeted Genes

Total RNA was extracted following standard protocol by Servicebio, Inc. (Wuhan Servicebio Technology Co., Ltd., Wuhan, Hubei, China). The primers used for PCR were designed with Primer Premier software (version 5.0; Premier Biosoft International, Palo Alto, CA, United States; primer sequence details are summarized in Table 2). cDNA synthesis was performed on a GeneAmp PCR System 9700 (Applied Biosystems; Thermo Fisher Scientific, Inc., Waltham, MA, United States) following the manufacturer’s instructions (RevertAid First Strand cDNA Synthesis kit, Cat.# K1622, Thermo Fisher Scientific, Inc., Waltham, MA, United States). qPCR was performed on a ViiA 7 Real-time PCR System (Applied Biosystems; Thermo Fisher Scientific, Inc.) using a PowerUp SYBR Green Master Mix (Cat.# A25778, Thermo Fisher Scientific, Inc.). The PCR thermal procedure is (1) 95°C, 10min, 2) 95°C, 15s– > 60°C, 60 s, 40 cycles. The fold change for each miRNA was calculated using the 2^–ΔΔCq^ method (Livak and Schmittgen, 2001). U6 expression level was used to normalize the mRNA expression data. Expression in six cell types (HFF (NC/SI/OE) and HSF (NC/SI/OE) were analyzed and triplicate experiments were done for all the cell types.

**TABLE 2:** RT-PCR primers.

**Gene RefSeq**	**Primer name**	**Primer Sequence(5′- > 3′)**
NM_001101	H-ACTIN-S	CACCCAGCACAATGAAGATCAAGAT
	H-ACTIN-A	CCAGTTTTTAAATCCTGAGTCAAGC
	U6-S	CTCGCTTCGGCAGCACA
	U6-A	AACGCTTCACGAATTTGCGT
	General control primer-A	TGGTGTCGTGGAGTCG
NM_000090.3	H-COL3A1-S	TTCCTTCGACTTCTCTCCAGCC
	H-COL3A1-A	CCCAGTGTGTTTCGTGCAACC
NM_000501.3	H-ELASTIN-S	GGCATTCCTACTTACGGGGTT
	H-ELASTIN-A	GCTTCGGGGGAAATGCCAAC
NM_212482.2	H-FN1-S	ACACAGAACTATGATGCCGACCA
	H-FN1-A	TGTCCATTCCCCACGACCAT
NM_003380.3	H-VIMENTIN-S	GAAGCCGAAAACACCCTGCAATC
	H-VIMENTIN-A	TGCAGCTCCTGGATTTCCTCT
NM_004612.3	H-TGFBR1-S	GGACCCTTCATTAGATCGCCCTT
	H-TGFBR1-A	CAACTTCTTCTCCCCGCCACT
NM_001145938.1	H-MMP1-S	TACGATTCGGGGAGAAGTGAT
	H-MMP1-A	AAGCCCATTTGGCAGTTGTG
NM_003254.2	H-TIMP1-S	TCCTGTTGTTGCTGTGGCTGAT
	H-TIMP1-A	AAACTCCTCGCTGCGGTTGT
NM_000088.3	H-COL1A1-S	CCAAGACGAAGACATCCCACCA
	H-COL1A1-A	CCGTTGTCGCAGACGCAGAT
MIMAT0000415	hsa-let-7i-5p-RT	CTCAACTGGTGTCGTGGAGTCGG CAATTCAGTTGAGAACAGCAC
	hsa-let-7i-5p-S	ACACTCCAGCTGGGTGAGGTAGT AGTTTGT

### Western Blotting

Whole protein lysate was extracted using RIPA Lysis buffer (Cat.#20101ES60, Yeasen, Shanghai, China) following instructions by the manufacturer. Equal concentration of protein was loaded on 5–10% SDS-PAGE gels and transferred onto a PVDF membrane (Cat.# IPVH000010, MilliporeSigma, Burlington, MA, United States). Primary antibodies for COPS6, COPS8, Ago1, Elf1,Tlr4, insulin-like growth factor 1 (somatomedin C) (IGF1), Collagen Type VIII (Collagen8), IL13, Bmp4 and tubulin were listed in Table 3. Bands were visualized using horse-radish peroxidase (HRP) conjugated secondary antibodies (Table 3) in conjunction with Immobilon ECL Ultra Western HRP Substrate (Cat.#WBKLS0100, MilliporeSigma, Burlington, MA, United States) via ImageQuant LAS 4000mini [HFF (NC/SI/OE) and HSF (NC/SI/OE)] were analyzed and triplicate experiments were done for all the cell types. AlphaEaseFC software (Genetic Technologies Inc., Miami, FL, United States) was used to analyze the density of electrophoretic Western blot bands by Servicebio, Inc. (Wuhan Servicebio Technology Co., Ltd., Wuhan, Hubei, China). GAPDH expression level was used to normalize the protein expression data. Intensity analysis was done for one of the experiments.

**TABLE 3:** Commercial antibodies.

**Antibody**	**Target protein**	**Provider**	**Catalog number**	**Dilution**
Peroxidase-Conjugated Goat anti-rabbit IgG (H + L)	Rabbit IgG (H + L)	Yeasen	33101ES60	1:5000
Peroxidase-Conjugated Goat anti-mouse IgG (H + L)	Mouse IgG	Yeasen	33201ES60	1:5000
Rabbit Anti-Goat IgG (H + L) HRP	Goat IgG (H + L)	Multisciences	70-RAG007	1:5000
Rabbit-COPS6 Polyclonal Antibody	COPS6	ABclonal	A7072	1:1000
Rabbit-anti-COPS8/COP9 (polyclonal)	COPS8/COP9	Proteintech	10089-2-AP	1:1000
Rabbit-anti-NEDD8 (polyclonal)	NEDD8	Proteintech	16777-1-AP	1:1000
Rabbit-anti-CUL1 (polyclonal)	Cullin-1	Proteintech	12895-1-AP	1:1000
Argonaute 1 (D84G10) XP Rabbit mAb #5053	Ago1	CST	5053T	1:1000
Rabbit-anti-ELF1 (polyclonal)	Elf1	Proteintech	22565-1-AP	1:1000
Mouse-anti-TLR4 (monoclonal)	Tlr4	Proteintech	66350-1-lg	1:1000
IGF1B-specific polyclonal antibody	Insulin-like growth factor 1	Proteintech	20215-1-AP	1:1000
Rabbit-anti-Collagen Type VIII (polyclonal)	Collagen Type VIII	Proteintech	17251-1-AP	1:1000
Rabbit-anti-IL13 (polyclonal)	IL13	SAB	38354	1:1000
Rabbit-anti-BMP4 (polyclonal)	BMP4	Proteintech	12492-1-AP	1:1000
Mouse-anti -beta Tubulin Mouse mAb	Tubulin	Servibebio	GB13017-2	1:1000

### Enzyme Linked Immunoabsorbent Assay (ELISA)

Supernatant of cell lysates was collected for each of six cell types [HFF (NC/SI/OE) and HSF (NC/SI/OE)], and the levels of MMP2, TGFβ1, and TIMP1 were quantified using ELISA kits as per manufacturers’ instructions (Human Matrix Metalloproteinase 2/Gelatinase A (MMP-2) ELISA Kit, Cat.#CSB-E04675h, CUSABio, Wuhan, Hubei, China; Human TGF-beta1 ELISA Kit, Cat.#EK1811, MultiSciences, Hangzhou, Zhejiang, China; Human TIMP1 ELISA Kit, Cat.#EK11382, MultiSciences).

### Statistical Analysis

Student *t*-test were performed for let-7i-5p expression comparison (unpaired, two tails, heteroscedastic) in six cell types (FNC, FSI, FOE, SNC, SSI, and SOE). ANOVA One Way analysis were also performed for validation (Supplementary File S1).

### Construction of the Let-7i-5p-Target Gene Regulatory Network and Functional Enrichment Analysis

miTarBase database (7.0) was used to predict the target genes of let-7i-5p^1^. The STRING database (www.string-db.org) was used to establish the protein-protein interaction (PPI) network. GO and KEGG pathway enrichment analyses were performed to determine the biological significance of associated proteins. Cytoscape version 3.7.2 was used to visualize the results.

## Results

### Let-7i-5p Regulates Cell Morphology and Motility in Normal and Pathogenic Fibroblasts

**FIGURE 1:**
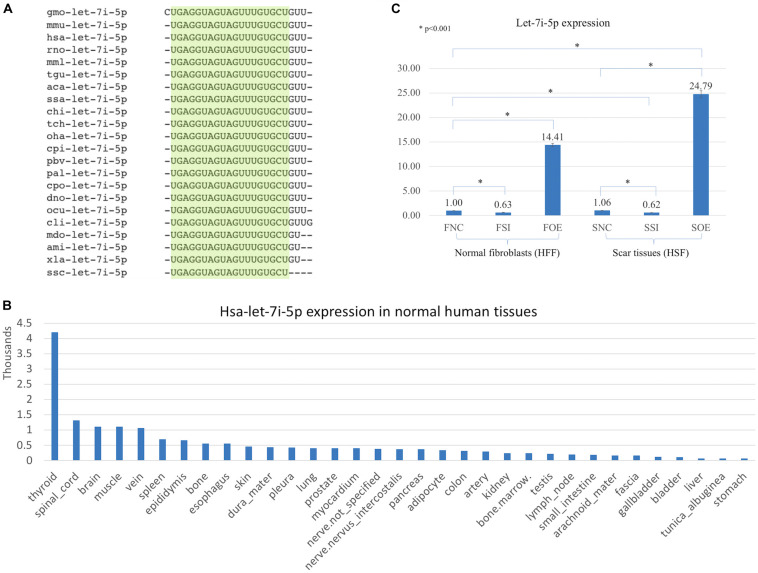
Let-7i-5p is conservative among different species and hsa-let-7i-5p is expressed differentially in normal human tissues. By lenti-viral infection the let-7i-5p expression was manipulated either up or down in normal and scar tissues. **(A)** Let-7i-5p sequence comparison across different species. The conservative sequences are highlighted. aca, Anolis carolinensis; ami, Alligator mississippiensis; chi, Capra hircus; cli, Columba livia; cpi, Chrysemys picta; cpo, Cavia porcellus; dno, Dasypus novemcinctus; gmo, Gadus morhua; hsa, Homo sapiens; mdo, Monodelphis domestica; mmL, Macaca mulatta; mmu, Mus musculus; ocu, Oryctolagus cuniculus; oha, Ophiophagus hannah; pal, Pteropus alecto; pbv, Python bivittatus; rno, Rattus Norvegicus; ssa, Salmo Salar; ssc, Sus scrofa; tch, Tupaia chinensis; tgu, Taeniopygia guttata; xla, Xenopus laevis. **(B)** Hsa-let-7i-5p expression levels in normal human tissues. Data based on two individuals’ microRNA sequencing results (Ludwig et al., 2016) and average of normalized value by quantile normalization were used. **(C)** let-7i-5p level was up- and down-regulated in normal and pathogenic fibroblasts by Lenti-viral transfection. *p < 0.001. F, normal fibroblasts (HFF). S, scar tissues. NC, non-transfected control. SI, transfected by lenti-KD miRNA to knock down hsa-let-7i-5p expression. OE, transfected by lenti-OE miRNA to overexpress hsa-let-7i-5p.

Let-7i-5p is a member of Lethal-7 (let-7) microRNA family, which is widely observed and highly conservative across species, from reptiles to mammals (Figure 1A). Let-7 family members were among the first discovered microRNAs and were shown to be an essential regulator of development in *C. elegans* (Reinhart et al., 2000), and let-7 microRNA family has been reported to regulate allergic inflammation through T cells (O’Connell et al., 2012). In human tissues, hsa-let-7i-5p showed extremely high expression in thyroid, and relatively high expression in spinal cord, brain, muscle and vein, with tissue specific index score at 0.905 (indicating a high tissue expression specificity in thyroid; Figure 1B; Ludwig et al., 2016), suggesting a potential role of hsa-let-7i-5p in metabolism, fibroblast proliferation and differentiation and tissue development. However, little is known about the role of let-7i-5p or the related molecular mechanism involved in normal fibroblast growth or fibrosis-related scar formation.

**FIGURE 2:**
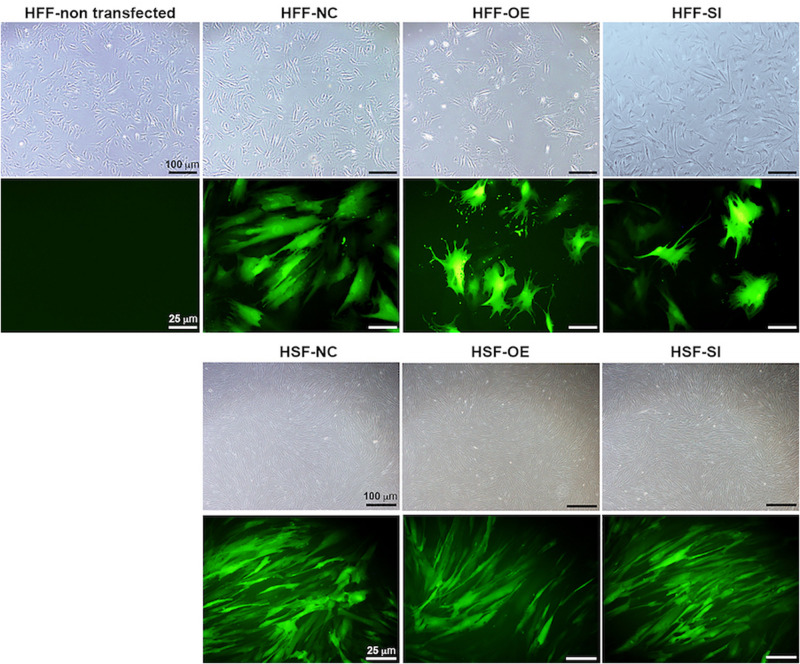
Let-7i-5p level change results in cell morphology changes in normal fibroblasts, but not in pathogenic fibroblasts. Scale bar in bright field images, 100 μm. Scale bar in fluorescent images, 25 μm.

Based on a recent miRNA profiling using PFUDD patients’ tissues, we found that let-7i-5p expression was really high in both normal and pathogenic fibroblasts based on miRNA sequencing. To manipulate the knock-down or overexpression of let-7i-5p, the miRNA level was up- and down-regulated in normal (HFF) and pathogenic (HSF) fibroblasts using Lenti-viral transfection and significant expression changes were observed (Figure 1C). Dysregulation of let-7i-5p in normal fibroblasts caused cell morphology changes yet had little influence on that of pathogenic fibroblasts (Figure 2). Surprisingly, either overexpression or knockdown of let-7i-5p resulted in rounder but more spiky cells. Similar phenotypes were reported to be caused by null-functional Dematin (an actin binding/bundling protein), and was associated with null effect in mutant fibroblasts and impaired wound healing process (Mohseni and Chishti, 2008).

**FIGURE 3:**
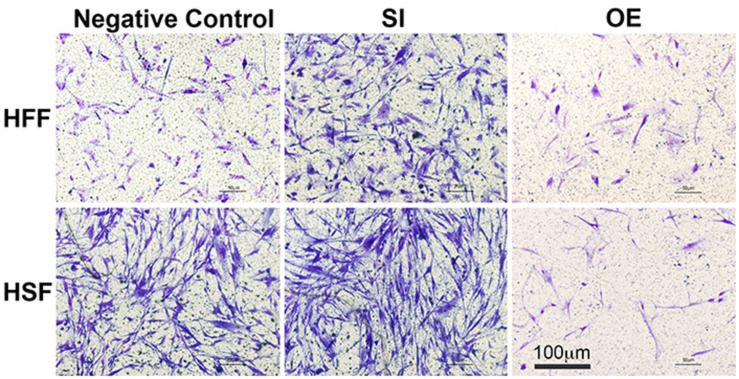
Overexpression of let-7i-5p could suppress migration, while inhibition could promote cell migration in both normal and pathogenic fibroblasts.

To see whether let-7i-5p could regulate cell motility, we performed cell migration assay for normal and pathogenic fibroblasts. Inhibition of let-7i-5p led to a clear promotion of cell motility, while overexpression of let-7i-5p displayed a severely suppression (Figure 3). The regulation patterns are similar in both normal and pathogenic fibroblasts (Figure 3).

### Let-7i-5p Regulates Cellular Processes Through Three Distinct Signaling Pathways

**FIGURE 4:**
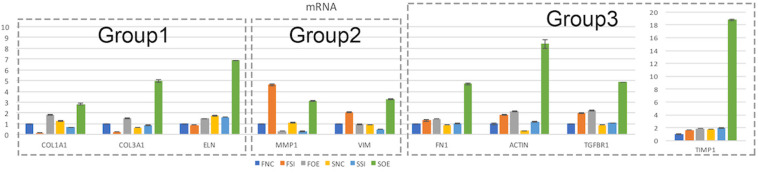
Let-7i-5p regulates signaling molecules in normal and pathogenic fibroblasts. Quantitative analysis of mRNA expression levels of let-7i-5p’s downstream targets were performed. Group 1, target genes positively regulated by hsa-let-7i-5p in normal fibroblast cells at mRNA level. Group 2, target genes negatively regulated by hsa-let-7i-5p in normal fibroblast cells at mRNA level. Group 3, target genes constitutively upregulated in normal and pathogenic fibroblasts at mRNA level with either suppressed or increased hsa-let-7i-5p expression. Error bar, standard error. mRNA level expression was evaluated by quantitative real-time PCR.

We then performed real time quantitative PCR to evaluate the mRNA expression of potential molecular regulators in those cellular processes. Interestingly, we found that up- or down-regulation of let-7i-5p results in three different regulatory patterns of those genes (Figure 4 and Table 4).

**TABLE 4:** mRNA expressions of let-7i-5p regulated targets.

	**Group 1**			**Group 2**		**Group 3**			
**mRNA**	**COL1A1**	**COL3A1**	**ELN**	**MMP1**	**VIM**	**FN1**	**ACTIN**	**TGBR1**	**TIMP1**
1 FNC	Control	Control	Control	Control	Control	Control	Control	Control	Control
2 FSI	Down	Down	Down	Up	Up	Up	Up	Up	Up
3 FOE	Up	Up	Up	Down	Down	Up	Up	Up	Up
4 SNC	Control	Control	Control	Control	Control	Control	Control	Control	Control
5 SSI	Down	Up	Down	Down	Down	Up	Up	Up	Up
6 SOE	Up	Up	Up	Up	Up	Up	Up	Up	Up

In the first group, let-7i-5p knockdown resulted in decreased mRNA expression of COL1A1, COL3A1, and ELN in normal fibroblasts, while overexpression of let-7i-5p resulted in significantly increased expression of those genes (Figure 4, Group 1). This suggests a positive correlation between let-7i-5p and those three genes. Pathogenic status (whether the cell is normal or pathogenic fibroblasts) doesn’t seem to affect this regulation, as the positive regulatory pattern is consistent in normal and fibrotic tissues for COL1A1 and ELN. The only exception of COL3A1 in let-7i-5p knockdown pathogenic fibroblasts, which had a slight increased expression instead of down regulation.

**FIGURE 5:**
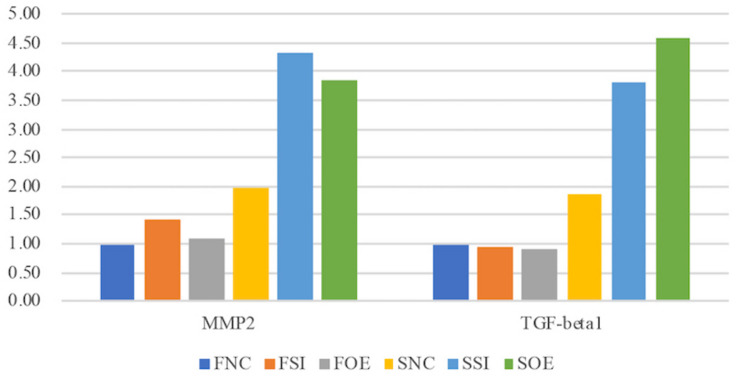
Let-7i-5p regulates MMP2 and TGF-beta1 proteins in normal and pathogenic fibroblasts (ELISA). Protein level expression was evaluated by ELISA.

In the second group, we observed quite opposite regulatory effects on mRNA expression of MMP1 and VIM by let-7i-5p (Figure 4, Group 2). Knockdown of let-7i-5p significantly enhanced MMP1 and VIM expression, while overexpression of let-7i-5p caused a decrease in expression. Strikingly, this negative regulation is completely reversed in pathogenic fibroblasts, as downregulation of let-7i-5p decreased the expression of MMP1 and VIM, and upregulation of let-7i-5p increased their expression. MMP2 expression displayed a similar negative pattern at protein level, although the expression level of MMP2 was almost doubled in control pathogenic fibroblasts (SNC) comparing to normal control cells (FNC) (Figure 5). This strongly suggests that regulation of MMP1 and VIM by let-7i-5p is dependent on the pathogenic status of the fibroblasts.

The third group of regulated genes contains FN1, ACTIN, TGFBR1, and TIMP1, which had increased expression with either knockdown or overexpression of let-7i-5p in both normal and pathogenic fibroblasts (Figure 4, Group 3). This suggests dysregulated let-7i-5p could boost up the expression of those target genes, regardless of the actual expression change of let-7i-5p (whether it is up- or down-regulated). It is worth noting that the enhanced expression was impressively high for all four target genes in fibrotic cells when let-7i-5p is over-expressed (Figure 4), suggesting that pathogenic fibroblasts could further amplify the regulatory effect of those genes resulted from let-7i-5p overexpression, while normal cells still maintained a retraining ability to suppress the dysregulation of target genes caused by let-7i-5p level changes. We also evaluated TGF-beta1, the ligand of TgfbR1, in corresponding cell types, and observed an opposite pattern of regulation in normal fibroblasts (Figure 5), which indicates a negative feedback regulation of TGF-beta in response to the TGFbetaR1 protein level changes in normal cells, and this regulation was lost in the pathogenic cells.

### Let-7i-5p Regulates Subcellular Functions in Normal Fibroblasts Through Direct Downstream Gene Targets

**FIGURE 6:**
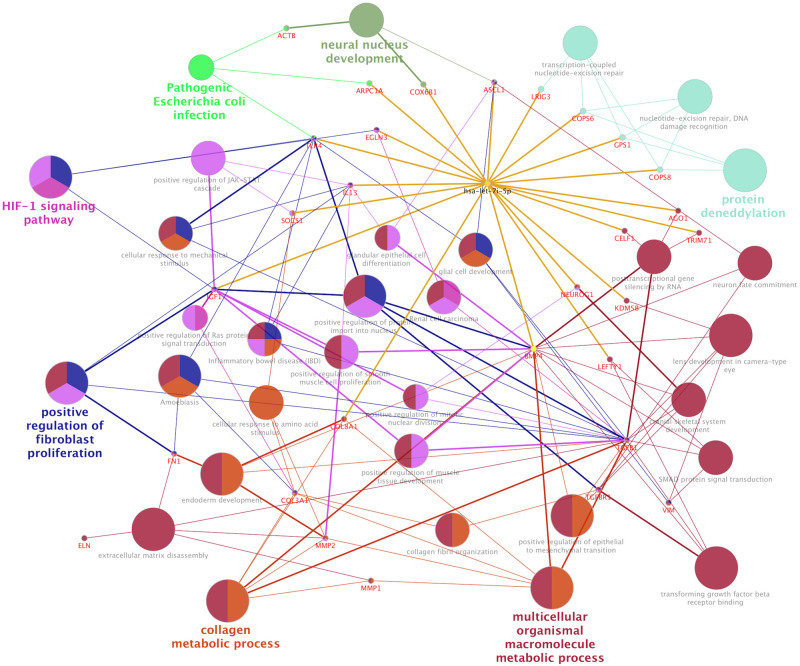
Let-7i-5p regulation mechanism is summarized in distinct functional pathways. Direct targets were connected to let-7i-5p by golden lines.

**FIGURE 7:**
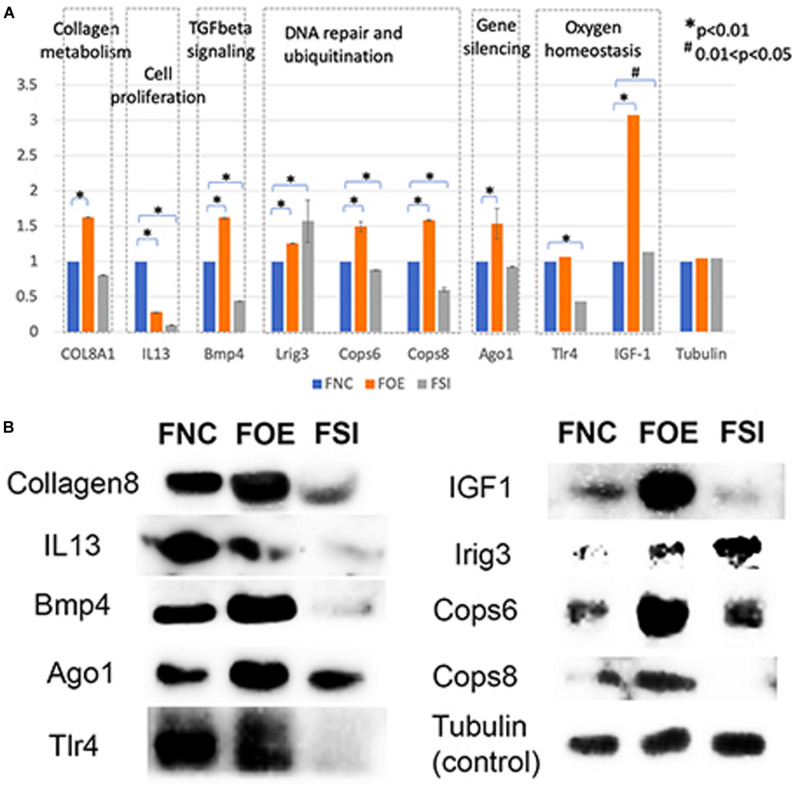
Let-7i-5p regulates direct target genes involved in collagen metabolism, cell proliferation, TGFbeta signaling, DNA repair and ubiquitination, gene silencing and oxygen homeostasis. **(A)** Quantitative analysis of protein expression levels of target genes. Y axis represents the expression change normalized by FNC for each protein. **(B)** Representative western blot images.

In normal human fibroblasts, manipulated let-7i-5p expression resulted in positive regulations of COL1A1, COL3A1 and ELN), negative regulations of MMP1 and VIM, and constitutively increased expression of FN1, ACTIN, TGFBR1, and TIMP1 at mRNA level. Given the fact that those molecules are involved in different signaling transduction pathways and corresponding subcellular functions, we performed functional enrichment analysis for those genes and let-7i-5p. We generated let-7i-5p centered signaling network based on protein-protein interaction and direct binding targets for let-7i-5p (Figure 6). Our data strongly suggest a multi-functional role of let-7i-5p in normal fibroblasts, including protein deneddylation, posttranscriptional gene silencing, oxygen homeostatic process (HIF-1 signaling), regulation of fibroblast proliferation, collagen metabolic process, pathogenic *E. coli* infection, neural nucleus development, extracellular matrix disassembly, etc. (Figure 6). Among the twenty direct targets predicted *in silico*, we found nine genes (COL8A1, IL13, BMP4, LRIG3, COPS6, COPS8, AGO1, TLR4, and IGF-1) which are predicted to interact with the molecules in the three signaling transduction pathways and might be serving as the connectors. To confirm let-7i-5p regulates through those direct downstream targets, we checked their expression in normal fibroblasts with up- or down-regulated let-7i-5p expression. We confirmed that let-7i-5p could directly regulate collagen metabolic process through COL8A1, fibroblast proliferation and epithelial cell differentiation through IL13, TGFbeta signaling through BMP4, DNA damage recognition, DNA repair and ubiquitination through LRIG3, COPS6 and COPS8, posttranscriptional gene silencing through AGO1 and ELF1 and oxygen homeostasis through TLR4 and IGF1 (Figure 7).

## Discussion

### Let-7i-5p Regulates Collagen Metabolic Process and Tissue Elasticity Through COL8A1, COL1A1, and COL3A1

The positive regulation of COL1A1, COL3A1, and ELN mRNA expressions in normal fibroblasts was largely retained in pathogenic cells, except for COL3A1, which has a slightly increased expression with decreased let-7i-5p. This indicates that the regulation of those target genes by let-7i-5p was not interrupted in pathogenic fibroblasts, or the functions of let-7i-5p in this specific signaling pathway is independent of the fibrotic status of cells. Since those proteins function together to strengthen and support connective tissues in the body, our data suggest an independent role of let-7i-5p in regulating collagen metabolism and tissue elasticity. This is in concordance with several studies elucidating the association between tissue elasticity and miRNA regulation. For example, in primitive neuroectodermal tumor (PNET) stem cells, tissue elasticity was suggested to promote miRNA silencing and downregulation of target genes (Vu et al., 2015). In mouse models, miR-29-3p could suppress the mRNA expression of COL1A1 and COL1A3 either with or without the induction by TGF-β1 and prevent *S. japonicum*-induced liver fibrosis (Tao et al., 2018). Also, Col1a1 and Col3a1 were overexpressed during active inflammation and murine colitis induced by 2,4,6-trinitrobenzene sulfonic acid (TNBS) hapten (Wu and Chakravarti, 2007). Given that COL8A1 is a direct target of let-7i-5p and it is regulated in the same positive pattern as that for COL1A1, COL1A3 in normal cells, our data suggest a positive regulation pattern of collagen metabolic process by let-7i-5p through COL8A1, COL1A1, and COL3A1 (Figures 4, 7).

### Let-7i-5p Regulates Extracellular Matrix (ECM) and Cell Migration Through MMP1, MMP2, and Vimentin

Let-7i-5p negatively regulated VIM and MMP1 in normal cells, and this regulation was interrupted in pathogenic fibroblasts, as their mRNA expressions were completely reversed from negative (in normal cells) to positive (in pathogenic cells) regulatory pattern (Figure 4). This observation indicates that let-7i-5p functions as an upstream regulator of Vim and MMP1 and its regulation is dependent on pathogenic status of the cells.

TGF-β1 and MMP2 protein levels were up-regulated, together with increased cell migratory capability in pathogenic fibroblasts comparing to normal cells (Figure 5). This is consistent with what was reported in human hepatic stellate cells, in which stimulation with TGF-β1 resulted in an increase in migratory capacity and up-regulated MMP-2 activity (Yang et al., 2003). However, in pathogenic fibroblasts with overexpressed let-7i-5p, the cell motility was decreased comparing to control pathogenic fibroblasts with no let-7i-5p change (Figure 3, lower right panel and lower left panel), while TGF-β1 and MMP2 expression levels were actually higher in let-7i-5p overexpressed pathogenic cells than that in control pathogenic cells (Figure 4). It is worth noting that IL13, a direct downstream target gene of let-7i-5p, could regulate smooth muscle cell proliferation together MMP2 (Figure 6), and it is regulated by let-7i-5p in a constitutively negative pattern (Figure 7), which is the opposite of that for TGFbR1 (Figures 4, 7).

### Let-7i-5p Regulates TGF-Beta Signaling and Fibroblast Proliferation Through BMP4, Fibronectin, Actin, TGFbetaR1, TIMP1, and IL13

The third group of targets regulated by let-7i-5p contains FN1, ACTIN, TGFBR1 and TIMP1, which are overexpressed at mRNA level upon dysregulation of let-7i-5p, no matter let-7i-5p’s expression is increased or decreased (Figure 4A). The consistent pattern in normal and pathogenic fibroblasts implies that the fibrotic status of cells doesn’t affect the regulation by let-7i-5p. Our data suggested that BMP4 is directly regulated by let-7i-5p, yet the regulation pattern is not similar to TGFBR1 or TGFbeta1 and let-7i-5p may regulate TGF-beta signaling in a parallel route that is independent from its regulation of BMP4.

Disruption of let-7i-5p regulation would result in morphological changes in normal fibroblasts (Figure 2), yet the mechanism remains unclear. In literature, let-7i-5p dysregulation phenotype mimics that of Dematin mutations (Mohseni and Chishti, 2008). Dematin is an actin binding/bundling protein that regulates FAK activation through RhoA and regulate cell morphology (Mohseni and Chishti, 2008) and is predicted to be a conserved target of miR181a-5p, miR181b-5p, miR181c-5p, miR181d-5p, and miR-4262 in human, yet little is known about its regulation by those miRNAs. In addition, it was reported that MMP1 overexpression could suppress Thioacetamide (TAA)-induced liver fibrosis in rat model (Iimuro et al., 2003). TIMP1’s function in fibrosis has been in argument as the evidences are divergent from different studies, although many of the results suggests that its expression has no effect on fibrosis (Giannandrea and Parks, 2014).

Interestingly, we found that dysregulated let-7i-5p could result in suppression of IL13, which is a positive regulator of fibroblast proliferation and epithelial cell differentiation (Figure 7). It may also play a role in JAK-STAT cascade and inflammatory response (Figure 6).

### Let-7i-5p and Its Potential Functions in DNA Repair and Ubiquitination, Gene Silencing and Oxygen Homeostasis

Hsa-let-7i-5p is predicted to target on several post-translational pathways, such as DNA repair and ubiquitination (through predicted target genes LRIG3, COPS6, COPS8, etc.) and gene silencing by RNA/miRNA (through target genes such as AGO1). It is also involved in homeostatic process [through predicted target genes TLR4 and IGF1, members of HIF-1 signaling pathway (Prabhakar and Semenza, 2015)] (Figure 6). Our data confirmed that let-7i-5p serves as a positive regulator of COPS6, COPS8, Ago1, and IGF-1 (Figure 7). LRIG3 is regulated in a quite different pattern comparing to COPS6 and COPS8, suggesting that let-7i-5p could regulate transcription-coupled nucleotide-excision repair (through LRIG3) separately from DNA damage recognition and protein deneddylation (through COPS6 and COPS8).

### Potential Clinical Applications of Hsa-let-7i-5p and MicroRNAs in PFUDD and Urological Diseases

miRNAs have been discussed as potential therapeutic targets and clinical biomarkers in various diseases (Mlcochova et al., 2015; Christopher et al., 2016; Ji et al., 2017). microRNAs are under investigation in a number of recent clinical trials for various urologic complications (e.g., urinary bladder neck obstruction, urolithiasis, urinary tract disorders, renal carcinoma, kidney injury, etc. (Table 5), mainly by microRNA profiling in patients, with a few extended into studies targeting on a specific microRNA (such as miR-21). A recent clinical trial (NCT02639923) evaluates the correlation between serum let-7i expression and intracranial traumatic lesions, which is based on evidence from animal models (Balakathiresan et al., 2012). The unique regulatory functions of let-7i-5p in fibroblast proliferation, ECM regulation and homeostasis makes it an interesting drug target for complications involved with fibrosis, tissue reconstruction and cellular stress (Figure 6). We hope the data from this study could broaden our understanding of the function of hsa-let-7i-5p in normal and pathogenic fibroblasts and urethral tissues, so as to facilitate clinical diagnosis, treatment as well as tissue engineering as follow-up options for patients with PFUDD or other urological diseases.

**TABLE 5:** miRNA associated clinical trials in urological diseases.

**NCT Number**	**Status**	**Study title**	**Conditions**	**Type**
NCT02470507	Active, not recruiting	Immune Function in Acute Kidney Injury	Acute Kidney Failure	General miRNA profile, observational study

NCT02289040	Completed	Acute Kidney Injury Following Paediatric Cardiac Surgery	Acute Kidney Injury	General miRNA profile, in microvesicles

NCT02315183	Completed	An Observational Case Control Study to Identify the Role of MV and MV Derived Micro-RNA in Post CArdiac Surgery AKI	Acute Kidney Injury	General miRNA profile, observational study

NCT03373786	Completed	A Study of RG-012 in Subjects With Alport Syndrome	Alport Syndrome	miR-21, renal

NCT00743054	Completed	microRNA Expression in Renal Cell Carcinoma	Carcinoma, Renal Cell	General miRNA profile, observational study

NCT03227055	Unknown	Cardiovascular Comorbidity in Children With Chronic Kidney Disease	Childhood Chronic Kidney Disease	urine exosome miRNA

NCT01114594	Completed	Pilot Study of RNA as a Biomarker for Autosomal Dominant Polycystic Kidney Disease	Chronic Kidney Disease Polycystic Kidney, Autosomal Dominant	General miRNA profile, urine, observational study

NCT02147782	Recruiting	Clinical Observation on Bone Metabolism Induced by Chronic Renal Insufficiency	Chronic Renal Insufficiency Renal Osteodystrophy	General miRNA profile, observational study

NCT02410876	Recruiting	Changes of microRNA Expression in Obstructive and Neurogenic Bladder Dysfunction	Disorder of the Lower Urinary Tract	General miRNA profile, comparison between BLUTD (bladder outlet obstruction (BOO)-induced) and NLUTD (neurogenic)

NCT00806650	Completed	Anti-IMP3 Autoantibody and MicroRNA Signature Blood Tests in Finding Metastasis in Patients With Localized or Metastatic Kidney Cancer	Kidney Cancer	General, miRNA profile, serum, observational study

NCT03089242	Unknown	MicroRNAs in Acute Kidney Injury	Kidney Injury in Cardiac Surgery - Expression of microRNAs	General miRNA profile

NCT01731158	Unknown	Sequential Therapy With Bevacizumab, RAd001 (Everolimus) and Tyrosinekinase Inhibitors (TKI) in Metastatic Renal Cell Carinoma (mRCC)	Metastatic Renal Cell Carcinoma	General miRNA profile

NCT03235128	Unknown	Clinical Significance of Assesment of Serum miRNA-30a in Childhood Nephrotic Syndrome	Nephrotic Syndrome Steroid-Resistant	miRNA-30a, serum, observational study

NCT00565903	Active, not recruiting	Elucidating the Genetic Basis of the Pleuropulmonary Blastoma (PPB) Familial Cancer Syndrome	Cystic Nephroma Pleuropulmonary Blastoma Sertoli-Leydig Cell Tumor of Ovary Medulloepithelioma Embryonal Rhabdomyosarcoma of Cervix Goiter Sarcoma Pineoblastoma Pituitary Tumors Wilms Tumor	General miRNA profile, observational study

NCT01482676	Completed	The Role of microRNAs in Organ Remodeling in Lower Urinary Tract Dysfunction	Urinary Bladder Neck Obstruction Cystitis, Interstitial Prostatic Hyperplasia	General miRNA profile, observational study

NCT02316522	Active, not recruiting	Epigenetic Contribution to the Pathogenesis of Diabetic Nephropathy in Qatari Population	Type 2 Diabetes	General miRNA profile, observational study

NCT01973088	Unknown	Screening and Identification of Human Urate Transporter hURAT1 MicroRNA	Urinary Calculi	miRNAs regulated by hURAT1

NCT03511924	Completed	Intradialytic Resistance Training in Haemodialysis Patients	Chronic Kidney Disease Requiring Chronic Dialysis	Renal specific miRNA profile

NCT03591367	Completed	The Potential Role Of MicroRNA-155 And Telomerase Reverse Transcriptase In Diagnosis Of Non-Muscle Invasive Bladder Cancer And Their Pathological Correlation	Bladder Cancer; Bladder Disease; Bladder Neoplasm; Micro-RNA	MicroRNAs-155

NCT04176276	Recruiting	Determining Serum and Urinary Levels of miRNA 192 and miRNA 25 in Patients With and Without Type 2 Diabetes.	Diabetic Kidney Disease; Type2 Diabetes	miR-192 and miR-25

NCT03924089	Recruiting	Oral Nutritional Supplement on Nutritional and Functional Status, and Biomarkers in Malnourished Hemodialysis Patients.	Malnutrition; End Stage Renal Disease	Circulating miRNAs

NCT01829971	Terminated	A Multicenter Phase I Study of MRX34, MicroRNA miR-RX34 Liposomal Injection	Primary Liver Cancer; SCLC; Lymphoma; Melanoma; Multiple Myeloma; Renal Cell Carcinoma; NSCLC	liposomal miR-34a mimic

NCT03942744	Recruiting	The Effect of High-flux Hemodialysis and On-line Hemodiafiltration on Endothelial Function.	Chronic Kidney Disease Requiring Chronic Dialysis	General miRNA profile

NCT04300387	Recruiting	Chronic Kidney Disease at Northeast Taiwan: Biomarker and Multidisciplinary Care	Chronic Kidney Disease	General miRNA profile

NCT02593526	Recruiting	Diuretic/Cool Dialysate Trial	Chronic Kidney Insufficiency	General miRNA profile

NCT03202212	Completed	Effect of Mixed On-line Hemodiafiltration on Circulating Markers of Inflammation and Vascular Dysfunction	Chronic Kidney Failure; Dialysis Related Complication	General miRNA profile in plasmatic exosomes or microvesicles

NCT03780101	Recruiting	Pathology and Imaging in Kidney Allografts	Renal Transplant Rejection; Chronic Kidney Diseases; Fibrosis	miR-214, miR-21 and miR-29

NCT03476460	Completed	Sodium Chloride and Contrast Nephropathy	Kidney Failure, Chronic; Kidney Failure, Acute; Heart Failure; Diabetes	General miRNA profile

NCT03844412	Suspended	Vestibulodynia: Understanding Pathophysiology and Determining Appropriate Treatments	Vestibulodynia; Temporomandibular Disorder; Fibromyalgia Syndrome; Irritable Bowel Syndrome; Migraines; Tension Headache; Endometriosis; Interstitial Cystitis; Back Pain; Chronic Fatigue Syndrome	General miRNA profile

NCT03651388	Completed	Research Into the Molecular Bases of a New Phenotype Combining Premature White Hair, Polycystic Kidney Disease, Aortic Dilation/Dissection and Lymphopenia	New Phenotype (Combining Premature White Hair, Polycystic Kidney Disease, Aortic Dilation/Dissection and Lymphopenia)	Bcl-2-regulating miRNAs

NCT03246191	Unknown status	Screening and Assessing the Risk Factors and Complications of Chronic Kidney Disease	Chronic Kidney Disease	General miRNA profile, circulating microRNA

NCT02691546	Unknown status	Screening for Chronic Kidney Disease (CKD) Among Older People Across Europe (SCOPE)	Chronic Kidney Diseases	General miRNA profile, circulating microRNA

## Conclusion

In this study, we analyzed let-7i-5p and its potential downstream targets (COL1A1, COL3A1, ELN, MMP1, VIM, FN1, ACTIN, TGFBR1, TIMP1, MMP2) in both normal and pathogenic fibroblasts. We found that let-7i-5p could regulate various signaling pathways and serve distinct functions in different cellular events, including tissue plasticity, cell motility and cell morphology. By functional enrichment analysis, we evaluated the potential direct targets of let-7i-5p that might be responsible for each signaling cascade. We conclude that let-7i-5p is a multi-functional regulator, and it could be affected by fibrosis and pathogenic status.

## Data Availability Statement

All datasets generated for this study are included in article/Supplementary Material.

## Ethics Statement

The experiments involving human participants were reviewed and approved by the Ethics Committee of Shanghai Sixth People’s Hospital. Consents were obtained from all of the patients to participate in this study.

## Author Contributions

KZ, JC, SZ, RY, and YW: clinical sample collection and experiments. KZ, XF, QF, and RC: experimental design. XF and EQ: data analysis. KZ, QF, and RC: reagents, materials, and analysis tools contribution. XF, KZ, QF, and RC: manuscript writing.

## Conflict of Interest

The authors declare that the research was conducted in the absence of any commercial or financial relationships that could be construed as a potential conflict of interest.

## Footnotes

1 http://mirtarbase.mbc.nctu.edu.tw/php/index.php

## Supplementary Material

FILE S1 Raw data used for Figures 1,4, and 7 and associated student *t*-test and ANOVA One Way analysis.
